# Scientific Basis for Use of *Pyrus pashia* Buch.-Ham. ex D. Don. Fruit in Gastrointestinal, Respiratory and Cardiovascular Ailments

**DOI:** 10.1371/journal.pone.0118605

**Published:** 2015-03-18

**Authors:** Khalid Hussain Janbaz, Muhammad Zaeem Ahsan, Fatima Saqib, Imran Imran, Muhammad Zia-Ul-Haq, Muhammad Abid Rashid, Hawa Z. E. Jaafar, Marius Moga

**Affiliations:** 1 Faculty of Pharmacy, Bahauddin Zakariya University, Multan, Pakistan; 2 The Patent Office, Karachi, Pakistan; 3 Department of Chemistry and Biochemistry, University of Agriculture, Faisalabad, Pakistan; 4 Department of Crop Science, Faculty of Agriculture, Selangor, Malaysia; 5 Department of Medicine, Transilvania University of Brasov, Brasov, Romania; Indian Institute of Integrative Medicine, INDIA

## Abstract

**Background:**

*Pyrus pashia* Buch.-Ham. ex D. Don. has been used conventionally by many communities in the Himalayan region for the management of gastrointestinal, respiratory, and vascular complications. Set against this background, this study was carried out to justify the scientific basis to validate folkloric uses of fruits of *Pyrus pashia* Buch.-Ham. ex D. Don. (Pp.Cr) in traditional systems of medicine.

**Methods:**

The crude ethanol extract of fruits of *Pyrus pashia* Buch.-Ham. ex D. Don. (Pp.Cr) was tested *in vitro* on isolated rabbit jejunum, tracheal, and aorta preparations. The responses of tissues were recorded using isotonic transducers coupled with a PowerLab data acquisition system.

**Results:**

The Pp.Cr on application (0.01–5.0 mg/ml) to isolated rabbit jejunum preparation exhibited relaxation through decrease in magnitude and frequency of spontaneous contractions. The Pp.Cr also exerted a relaxant (0.01–5.0 mg/ml) effect on K+(80 mM) induced contractions in isolated rabbit jejunum preparations and caused shifting of the Ca2+ curves (1.0–3.0 mg/ml) toward right in a manner similar to that of verapamil (3μM), possibly suggesting presence of Ca2+ channel blocking activity. Subsequently, Pp.Cr in a concentration-dependent fashion (0.01–10.0 mg/ml) caused relaxation of CCh (1μM) and K+ (80 mM) induced contractions in isolated rabbit tracheal preparations in a manner comparable to that of dicyclomine, suggesting that the observed relaxant effect is likely to be mediated through antimuscarinic and/or Ca2+ channel blocking activities. Moreover, when evaluated against isolated rabbit aortic preparations, the Pp.Cr in concentrations up to 10 mg/ml exhibited a contractile response that was found to be abolished subsequent to pretreatment of isolated tissue preparation with cyproheptadine (1μM), phentolamine (1μM), and losartan (1μM), suggesting that Pp.Cr may have some α-adrenergic, muscarinic, serotonergic, and angiotensin II activities.

**Conclusions:**

The aqueous ethanolic extract of *Pyrus pashia* (Pp.Cr) exhibited spasmolytic, bronchodilator, and vaso-constrictive activities possibly through different mechanisms. The spasmolytic and bronchodilator activities are likely to be mediated through blockade of Ca2+ channels, while vasoconstrictive activity may be due to presence of a α-adrenergic, muscarinic, serotonergic, and angiotensin II agonistic component.

## Introduction

*Pyrus pashia* Buch.-Ham. ex D. Don. (*Rosaceae*), is a medium size fruiting tree, known locally as Indian pear, Himalayan pear, Batangi, Tangai and Mehal [1,2]. In Pakistan, it is distributed widely in Chitral, Swat, Kaghan, Hazara, Muree, Poonch, Mirpur districts at elevations between 750 and 2500 m. The plant is well-known for its nutritional and therapeutic importance.

The leaves are bitter in taste, served as fodder for goats and sheep [3] as well as butter tea beverages by the *Monpa* community of Tawang, Arunachal Pradesh,India [4]. The fresh leaves are known to possess astringent, febrifuge, laxative and sedative properties and crushed leaves are used to improve cosmetic appearance by staining palms, feet and nails [5].The fruits are tasty and eaten by local people as diet and are known to be useful in constipation [6] while fruit is used to minimize thirst. Fruit juice is astringent and diuretic [7], and is used to manage dysentery [8], leishmaniasis [9], eye problems [10], digestive disorder, sore throat, irritability, abdominal pain, anemia [11].Lab investigations have proved antimicrobial, antioxidant, stomachic and hypoglycemic activities of fruit [12,13]. Decoctions containing dried fruits with other plant parts are used for improvement in spleen and stomach functions [14]. The fruit is added to cattle fodder to enhance milk production [15]. The barks of tree possess astringent, laxative, anthelmintics and febrifuge properties [16] and is used traditionally to manage digestive disorders [17–19]. The barks possess astringent and tonic properties and are used to manage sore throat, fever, peptic ulcer, gastric ulcer [20–25] and typhoid fever [18].

Phytochemical and pharmacological investigations of fruits revealed the presence of secondary metabolites like alkaloids, flavonoids, steroids, and tannins [26], lupeol, β-sitosterol, β-sitosterol—β-D-glucoside [9] and possesses antimicrobial activity against *Klebsiella pneumonia*, *Shigella flexneri* and *Eschericia coli* [26].Leaves has been known to contain arbutin, tannins, phlorhizin, pectin and amygdalin [27], chlorogenic acids, flavan-3-ols and arbutin, reported to possess antioxidant, anti-mutagenic and anti-carcinogenic activities [28–30]. Although used as a remedy for multiple ailments like airway and gastric and cardiovascular ailment, *Pyrus pashia* Buch.-Ham. ex D. Don is a very less-researched plant, as no detailed pharmacological studies exists on this plant. As part of series of experiments in our laboratory on validating tagged biological and physiological activities of medicinal plants [31–40] the current study was designed to investigate and validate the therapeutic potential of *Pyrus pashia* Buch.-Ham. ex D. Don in cardiovascular, respiratory and gastrointestinal ailments.

## Materials and Methods

### Collection of plant material and extraction

The fresh ripened fruits of *Pyrus pashia* Buch.-Ham. ex D. Don were collected from Abbottabad, Pakistan. No specific permissions were required for these locations/activities and we did not sample any endangered species. The plant material was got authenticated by kind cooperation of an expert taxonomist Professor Dr. Altaf Ahmad Dasti, at the Institute of Pure and Applied Biology, Bahauddin Zakariya University, Multan vide voucher numbers P. Fl. 364–7. The plant material was made free from contaminating materials adulterants and grinded to a coarse powder with the help of a special herbal grinder. The coarsely grinded herbal material was extracted by triple maceration procedure and about 1 Kg material was macerated with 70% aqueous-ethanol at room temperature for 1 week in amber colored container with occasional shaking. The soaked material was passed through muslin cloth to remove the organic debris and the fluid obtained was filtered through Whatmann-1 filter paper. The filtrates obtained following successive macerations were pooled and was evaporated under reduced pressure in a rotary evaporator (Buchi R-200 Switzerland) attached with re-circulating chiller (B-740) and vacuum pump (Buchivac V-500) at 37°C to a thick paste of semi solid consistency. The obtained crude extract was stored at -4°C in air tight jars. The approximate percentage yield of the crude extract was calculated to be 16.5%. The crude ethanolic extract of *Pyrus pashia* (Pp.Cr) was insoluble in distilled water and was rendered soluble by means of Tween 80 to prepare stock solution of 300 mg/ml, which was subjected to series of dilutions in normal saline to make 3mg/ml concentrations on the day of experiment. The final diluted vehicle was found without any effect on tissue response in control experiments.

### Drugs and Chemicals

Acetylcholine chloride, carbachol, potassium chloride, verapamil hydrochloride and phenylephrine, magnesium chloride, ethylene tetra-acetic acid (EDTA) were purchased from Sigma Chemicals Co. St Louis, MO, USA. Calcium chloride, glucose, magnesium sulphate, potassium dihydrogen phosphate, sodium bicarbonate, sodium dihydrogen phosphate, and ethanol were obtained from Merck, Darmstadt, Germany. Ammonium hydroxide, sodium chloride, and sodium hydroxide were purchased from BDH Laboratory supplies, Poole, England.

The chemicals used in these experiments were of highest purity and reagent analytical research grade. Stock solutions and subsequent dilutions were made fresh in distilled water on the day of experiment. The drugs were made soluble in vehicles which were without any effect on tissues in control experiments.

### Phytochemical screening

The crude ethanol extract of Pyrus pashia (Pp.Cr) was subjected to for qualitative phytochemical analysis for the possible presence of alkaloids, saponins, anthraquinones, coumarins, sterols, terpenes, flavonoids and phenols [41].

### Experimental animals and their housing condition

Animals (♂/♀) used in this study were local strain rabbits (1.0–1.8 kg) purchased from the local market with age limit between 6–7 months. These were housed under controlled environmental condition (23–25°C) at the animal house of Faculty of Pharmacy, Bahauddin Zakariya University, Multan. The animals were provided with standard food and tap water *ad libitum*. The animals were deprived of food 24 hr prior to the experiments but were given free access to water. Rabbits were sacrificed following a blow on back of head to be used for *in vitro* studies. All the experiments performed complied with the rulings of Institute of Laboratory Animal Resources, Commission on Life Sciences [42] and approved by the Ethical Committee of Bahauddin Zakariya University, Multan (EC/12/2011 dated 16.02.2011).

### *In vitro* experiments

*In vitro* experiments were performed following minor modification of the reported methods [31–34]. Briefly, tissue segments of jejunum, trachea and aorta from the rabbit were prepared and maintained adequately in the respective buffer solutions. The detailed explanation of each tissue extraction procedure is described below under the heading of tissue of interest.

### Isolated rabbit jejunum preparations

The crude ethanolic extract of *Pyrus pashia* (Pp.Cr) was tested for the possible presence of either spasmolytic or spasmogenic activity by using isolated rabbit jejunum preparations. Isolated rabbit jejunum segments of approximately 2 cm in length were suspended in isolated tissue baths containing Tyrode’s solution, at 37°C, aerated with carbogen (95% O_2_ and 5% CO_2_). The composition of the Tyrode’s solution (mM) was: KCl (2.68), NaCl (136.9), MgCl_2_ (1.05), NaHCO_3_ (11.90), NaH_2_PO_4_ (0.42), CaCl_2_ (1.8) and glucose (5.55). A preload of 1 gm was applied and intestinal responses were recorded through isotonic transducer by Power Lab Data Acquisition System (AD Instruments, Sydney, Australia) attached to a computer installed with Lab Chart Software (Version 6). The tissues were allowed to equilibrate for at least 30 min. prior to the addition of any drug. Isolated rabbit jejunum preparations exhibit spontaneous rhythmic contractions and allow testing of the antispasmodic (relaxant) effect without application of an agonist [32,43]. The observed response of the test material was quantified by the application of doses in a cumulative fashion. The relaxant effects on the part of test substances were taken as the percent change in spontaneous contractions of the preparation recorded immediately before the addition of test substances.

The possible mechanism of the relaxant activity of the test materials were investigated through the relaxation of the observed sustained spasmodic contractions following exposure to high concentration of K^+^(80 mM) [44]. The test materials were applied in a cumulative manner to the sustained contractions to achieve concentration-dependent inhibitory responses [45]. The observed relaxant effect of the test materials on K^+^ (80 mM)-induced contraction was expressed as percent of the control contractile response.

Calcium channel blocking effect of the test substances were confirmed by the method described previously by Gilani *et al* [46]. The isolated rabbit jejunum preparations were allowed to stabilize in normal Tyrode’s solution, which were subsequently replaced for 30 min with Ca^2+^-free Tyrode’s solution to which EDTA (0.1 mM) was added in order to remove calcium from the tissues. This bath solution was further replaced with K^+^-rich and Ca^2+^-free Tyrode’s solution, having the following composition (mM): KCl (50), NaCl (91.04), MgCl_2_ (1.05), NaHCO_3_ (11.90), NaH_2_PO_4_ (0.42), glucose (5.55) and EDTA (0.1). Subsequent to an incubation period of 30 min., cumulative Ca^2+^ concentrations were applied to the tissue bath to obtain control calcium dose-response curves (DRCs). On achievement of the super-imposable control calcium dose-response curves (usually after two cycles), the tissues were then washed and allowed to equilibrated with the plant extract for 1 hr and then the concentration response curves of Ca^2+^ were recorded and compared to the control curves. The DRCs of Ca^2+^ were recorded in the presence of different concentrations of the plant extracts in tissue bath.

### Isolated rabbit tracheal preparations

The rabbit tracheas were dissected out and kept in Krebs solution of the following composition (mM): NaCl (118.2), NaHCO_3_ (25.0), CaCl_2_ (2.5), KCl (4.7), KH_2_PO_4_ (1.3), MgSO_4_ (1.2) and glucose (11.7). The trachea was cleaned free from the surrounding fatty tissues and rings of 2–3 mm width containing 2–3 cartilages were prepared. Each ring was opened by a longitudinal incision on the ventral side opposite to the smooth muscles layer to form a strip with smooth muscles layer in middle and cartilages on both sides. These tracheal preparations were mounted in 20 ml organ bath containing Krebs solution being maintained at 37°C and aerated with carbogen. A preload tension of 1g was applied and tissue preparations were allowed to be equilibrated for 1 hour prior to any challenge by the drug. Tissue preparations were stabilized by repeated applications of carbachol (1μM) until constant responses were recorded. The carbachol (1μM)- and high K^+^(80 mM)-induced sustained contractions were subsequently used for testing of different doses of the test material in a cumulative fashions. The isometric responses were recorded through Power Lab Data Acquisition System (AD Instruments, Sydney, Australia) attached to a computer installed with Lab Chart Software (Version 6). The standard drug with Ca^+2^ channel blocking effect (verapamil) was tested on high K^+^(80 mM)- and carbachol- induced spastic contractions in order to confirm the possible mechanism of action.

### Isolated rabbit aorta preparation

The effect of Pp.Cr on systemic vascular resistance was assessed on isolated rabbit aorta preparations. Rabbits of either sex were sacrificed by a blow on the back of head and descending thoracic aorta was dissected out and kept in the normal Krebs solution having composition as described earlier. It was then cut vertically in 2–3mm width segments. Each isolated tissue segment was then hung in tissue organ bath (Radnoti) containing Kreb’s solution aerated with carbogen (95% oxygen and 5% carbon dioxide) at temperature 37 C. A pre-load of 2 g was applied to each preparation and allowed to equilibrate for a period of 1 hr. After equilibration, tissue was stabilized by repeated exposure to K^+^ (80mM) or phenylephrine (1μM) depending upon the protocol of the experiment. The vasorelaxant /vasoconstrictive effects of the test substances were studied by addition in tissue organ baths containing pre-stabilized tissue in a cumulative manner. Changes in isometric tension of aortic rings were obtained via force-displacement transducer (Model FORT100, WPI, USA) coupled to Power Lab data acquisition system (AD Instruments, Sydney, Australia) and computer running Lab Chart software (version 6).

### Statistical analysis

The data is expressed as mean ± S.E.M. (n = 5) and median effective concentration (EC_50_) are given with 95% confidence intervals (CI) and the logarithmic dose response curves of different treatments were then plotted using Computer software “Graphpad Prism” (Graph Pad Software, San Diego, CA, USA).

## Results

### Preliminary phytochemical analysis

The Pp.Cr was subjected to preliminary phytochemical analysis to detect presence of the members of different phytochemical groups (i.e., alkaloids, tannins, saponins, coumarins, anthraquinones, sterols, flavonoids and terpenes) and results obtained indicated the presence of alkaloid, saponins, tannins, terpinoids, anthraquinones and flavonoids as ethanol soluble extractable constituents.

### Effect on isolated rabbit jejunum preparations

**Fig 1:**
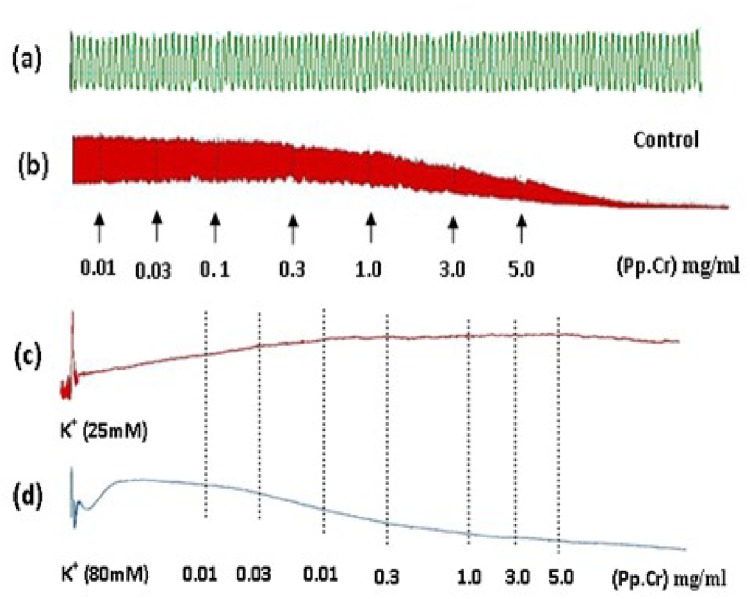
Original tracings presenting (a) spontaneous contraction of isolated rabbit jejunum in controlled biological environment and spasmolytic effect mediated by crude ethanolic extract of *Pyrus pashia* (Pp.Cr) on (b) spontaneous (c) low K^+^ (25 mM) and (d) high K^+^ (80 mM) induced tissue contraction. Pp.Cr was added in increasing concentrations and values listed were the final tissue bath concentrations. Each tracing is a presentation of single jejunum used.

**Fig 2:**
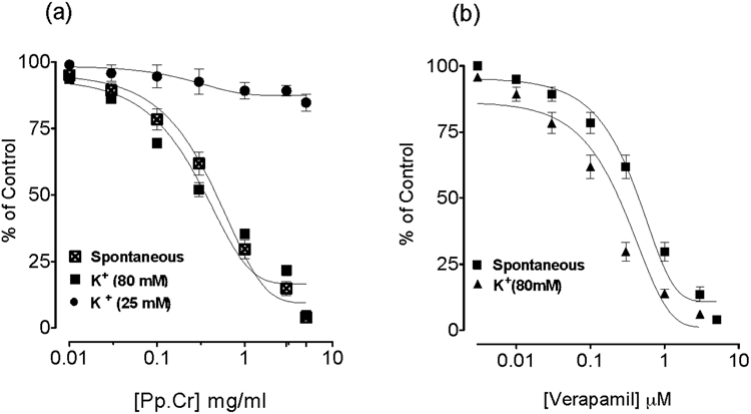
Spasmolytic effect observed in concentration-dependent manner by (a) a ethanolic extract of *Pyrus pashia* (Pp.Cr) and (b) verapamil on spontaneous- and high K+ (80 mM)-induced contractions in isolated rabbit jejunum preparations. Values are the mean ± SEM, n = 5.

The Pp.Cr exerted relaxant effect on spontaneous contractions in isolated rabbit jejunum preparations in a manner proportional to concentration at isolated tissue bath, in concentration range of 0.01–5.0 mg/ml with EC_50_ value of 0.5131 mg/ml (95% CI: 0.3815–0.6902 mg/ml; n = 5) (Figs. 1b and 2a).

**Fig 3:**
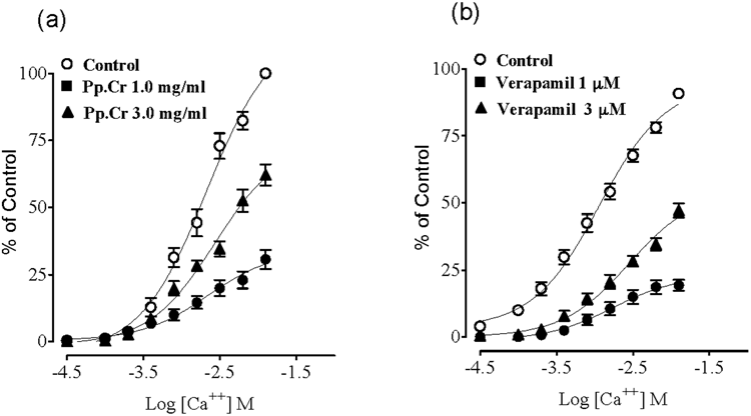
Calcium antagonizing effect of (a) ethanolic extract of *Pyrus pashia* (Pp.Cr) and (b) verapamil on concentration response curves of Ca^2+^ in isolated rabbit jejunum preparations. Values are the mean ± SEM, n = 5. Control CaCl_2_.

Pp.Cr was unable to relax K^+^ (25 mM)-induced spastic contractions (Fig. 1c and Fig. 2a), but exhibited complete relaxation of K^+^(80mM)-induced contractions in isolated rabbit preparations with EC_50_value of 0.3066 mg/ml (95% CI: 0.2226–0.4221 mg/ml; n = 5) (Figs. 1d and 2a). Verapamil relaxed the spontaneous and high K^+^ (80 mM) induced contractions with EC_50_ values of 0.1623 μM (95% CI: 0.1262–0.2086 μM; n = 5) and 0.1984 μM (95% CI: 0.1435–0.2744μM; n = 5) respectively (Fig. 2b). Moreover, pretreatment of the isolated rabbit jejunum preparations with Pp.Cr caused a rightward shift of concentration response curves for Ca^2+^ in a manner similar to that of verapamil (Fig. 3).

### Effect on isolated rabbit tracheal preparations

**Fig 4:**
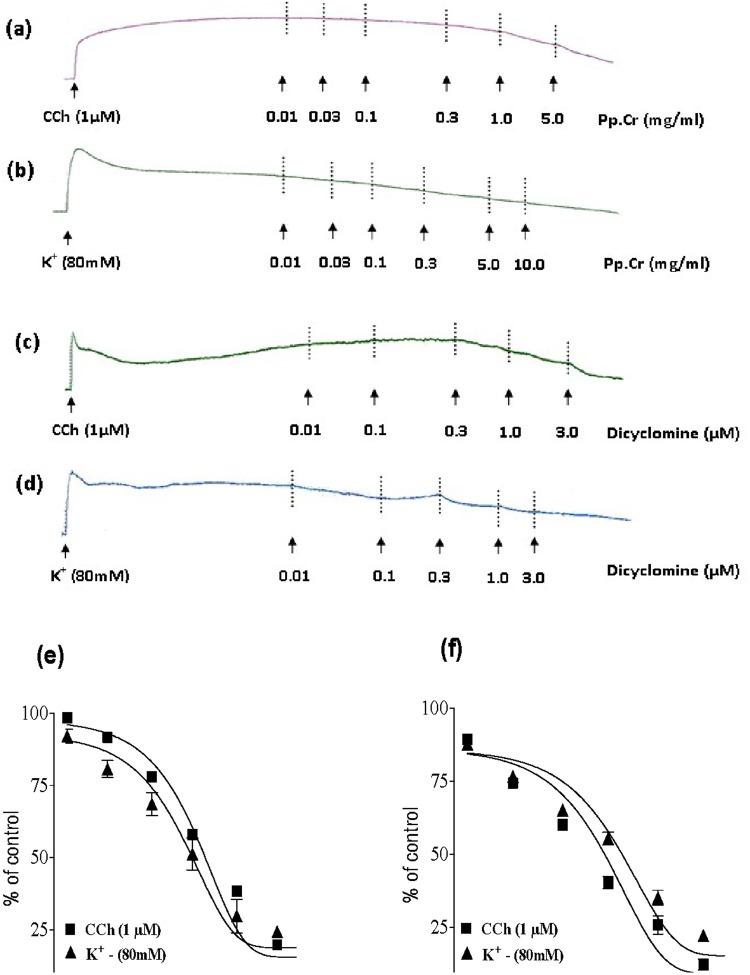
Concentration dependent broncho-relaxant effect of a ethanolic extract of *Pyrus pashia* (Pp.Cr) (a, b and e) and dicyclomine (c,d and f) on carbachol (CCh: 1μM)- and high K^+^ (80 mM)- induced contractions in isolated rabbit tracheal preparations. Values are the mean ± SEM, n = 5.

The Pp.Cr exerted relaxant effect on carbachol (CCh; 1 μM) and K^+^ (80 mM) induced contractions in isolated rabbit tracheal preparations with respective EC_50_ value of 0.5096 mg/ml (95% CI: 0.4046–0.635 mg/ml; n = 5) (Fig. 4) and 0.3839 mg/ml (95% CI: 0.2884–0.5111 mg/ml; n = 5) (Fig. 4).

Comparison of the EC_50_ values of Pp.Cr for CCh (1μM) and K^+^(80 mM)-induced contractions pointed out lower numerical value of EC_50_ of Pp.Cr for K^+^ (80 mM) induced contractions than CCh -induced contractions, suggesting that Pp.Cr was more effective on K^+^ (80 mM)-induced contractions than on CCh-induced contractions. Similarly dicyclomine caused relaxation of CCh (1 μM) and K^+^(80 mM)-induced contractions with EC_50_ values of 0.1673 mg/ml (95% CI: 0.1280–0.2186 mg/ml; n = 5) and 0.3937 mg/ml (95% CI: 0.2728–0.5682 mg/ml, n = 5) (Fig. 4) respectively.

### Effect on isolated rabbit aorta preparations

**Fig 5:**
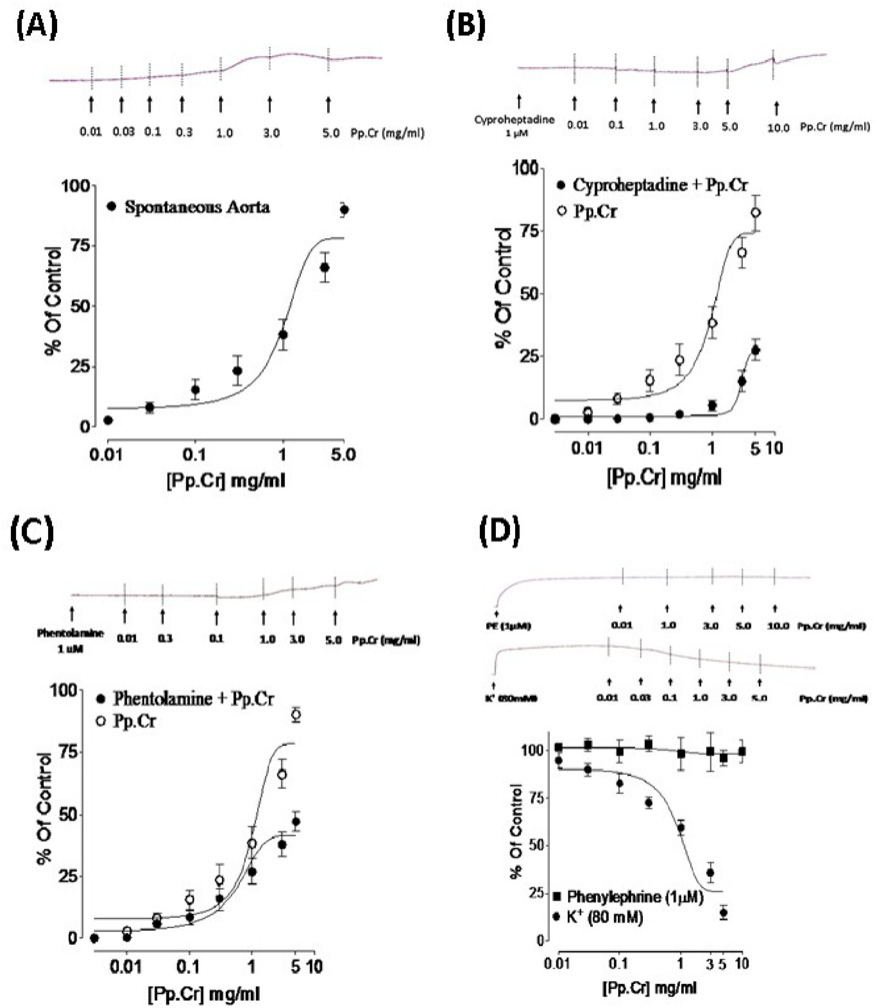
Tracings showing (A) Spasmogenic effect of *Pyrus pashia* (Pp.Cr) in concentration dependent manner on spontaneously contracting isolated rabbit aortic tissue. Further, pretreatment of aortic tissue with each 1μM of cyproheptadine (B) and phentolamine (C) significantly inhibited the spasmogenic effect of Pp.Cr. When isolated aortic tissue was pre-contracted with phenylephrine (PE: 1μM)- and high K^+^ (80 mM), *Pyrus pashia* (Pp.Cr) exhibited concentration dependent vasodilator effect against high K^+^ while ineffective against phenylephrine (D). Values are the mean ± SEM, n = 5.

The Pp.Cr on application to the isolated rabbit aortic preparations exerted tissue bath concentration (0.3–5.0 mg/ml) dependent contractile response (Fig. 5A).

The percentage contractile responses corresponding to respective tissue bath concentrations of 0.01, 0.03, 0.1, 0.3, 1.0, 3.0 and 5.0 mg/ml were recorded to be 2.786 ± 0.16, 8.093 ± 0.73, 15.524 ± 0.56, 23.0498 ± 0.97, 38.236 ± 1.08, 66.105 + 1.631 and 90.00 ± 1.429. The Pp.Cr-induced contractile response was found to be decreased on pretreatment of tissues with cyproheptadine (1 μM) (Fig. 5B), phentolamine (1 μM) (Fig. 5C) and losartan (figure not shown). Furthermore, Pp.Cr did not relax phenylephrine (1 μM)-induced contractions in isolated rabbit aortic preparation (Fig. 5D) but relaxed the K^+^ (80mM)-induced contraction at tissue bath concentration range of 0.1–5 mg/ml with EC_50_ value of 0.5515 mg/ml (95% CI: 0.2602–1.169 mg/ml; n = 5) (Fig. 5D).

## Discussion

**Fig 6:**
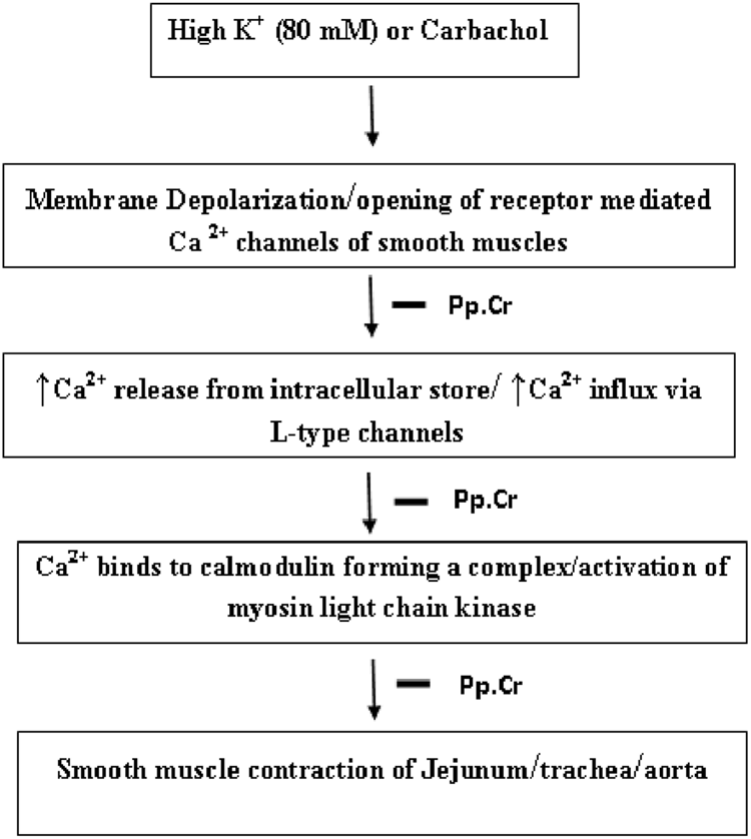
Proposed mechanism of action of smooth muscle relaxation mediated by ethanol extract of *Pyrus* pashia (Pp.Cr) against high K^+^ (80 mM) and carbachol (1μM) induced contraction. Sign—indicates site of inhibition by Pp.Cr.

*Pyrus pashia* has folkloric repute for use in the management of gastrointestinal disorders in traditional systems of medicine, hence, Pp.Cr was subjected to a battery of tests to validate its use in native systems of medicine. The Pp.Cr on application to the spontaneous contractions in isolated rabbit jejunum preparations, suppressed the contractions in terms of magnitude and frequency. The spontaneous contractions in jejunum are function of periodic depolarization and repolarization, while action potential is generated through rapid influx of Ca^+2^ via the voltage dependent L channels (VDLCs) at the time of maximal depolarization [47]. The suppression of spontaneous movements in jejunum by Pp.Cr is presumed to be mediated through blockade of Ca^+2^ channels or alternatively via opening of K^+^ channels [32–33], which was concluded further by testing Pp.Cr on K^+^(25 mM)- and K^+^ (80 mM)-induced contractions in isolated rabbit jejunum preparations. The Pp.Cr did not cause much relaxation of K^+^(25 mM)-induced contractions, indicated that relaxant activity on the part of Pp.Cr was not mediated through opening of K^+^ channels. Alternatively, addition of Pp.Cr to tissue baths in cumulative manner completely relaxed the K^+^(80 mM)-induced contractions, suggested that the observed relaxant activity was likely to be mediated through blockade of Ca^2+^ channels. The contractile elements in smooth muscle preparations, i.e., rabbit jejunum, trachea and aorta are activated through increase in cytoplasmic free Ca^2+^ concentration via opening of the voltage dependent L-type Ca^2+^ channels (VDLCs) [48–49] or release of Ca^2+^ from sarcoplasmic stores [50]. The Pp.Cr exerted relaxant effect through blockade of Ca^+2^ channels and further inhibition of sequence of events, i.e., decrease in cystolic Ca^2+^ concentration, decrease in Ca^2+^ binding to calmodulin, decrease in Ca^2+^ calmodulin complex formation, decrease in activation of myosin light chain kinase (MLCK), decrease in phosphorylation of the myosine light chains, decrease in interaction between actin and myosin and inhibition of contractile phenomenon (Fig. 6). These speculations were confirmed further as pre-treatment of isolated rabbit jejunum preparations with Pp.Cr caused rightward shift of Ca^2+^ response curve in a manner similar to verapamil as a standard Ca^2+^ channel blocker [51]. The Ca^2+^ channel blockers are a class of therapeutic agents being effective in the management of hyperactive gut diseases. The bronchodilator potential of Pp.Cr was explored following application to carbachol (1 μM) and K^+^ (80 mM)-induced contractions in isolated rabbit tracheal preparations. The Pp.Cr addition to isolated tissue bath in cumulative manner, caused a concentration dependent relaxation of both CCh (1μM)- and K^+^(80 mM)-induced contractions. However, relaxant effect of Pp.Cr on K^+^(80 mM)-induced contractions was achieved at much lower tissue bath concentrations as compared to carbachol (1 μM)-induced contractions. The carbachol is one of the cholinergic drugs, causing increase in cytosolic Ca^+2^ concentrations. On the basis of above-mentioned arguments, presence of both activities, i.e., anti-muscarinic and blockade of Ca^2+^ channels was speculated in a manner similar to dicyclomine (Fig. 6).

Moreover, Pp.Cr exhibited contractile activity on isolated rabbit aortic preparation in tissue bath concentration range of 0.1–5 mg/ml, which was found to be minimized on pre-treatment of isolated rabbit aortic preparations with phentolamine, cyproheptadine and losartin and was concluded to be mediated through activation of multiple receptors like α-adrenergic, muscarininic, histaminergic, serotonergic and angiotensin II receptors [52]. The Pp.Cr also caused relaxation on K^+^ (80 mM)-induced contractions in isolated rabbit aortic preparations in a manner similar to isolated rabbit jejunum and rabbit tracheal preparations, hence Ca^2+^ channel blocking activity of Pp.Cr was confirmed in all types of smooth muscles preparations.

The Pp.Cr exhibited Ca^2+^ channel blocking activity in isolated rabbit tissue preparations (i.e., jejunum, trachea and aorta) which can be attributed to the presence of alkaloids and flavonoids among the constituents of *Pyrus pashia* detected in preliminary phytochemical screening. The phenylephrine-induced contractions in isolated rabbit aortic preparations were not relaxed on application of Pp.Cr because it involves activation of α-adrenergic receptors, activation of G-proteins, increase in Ca^2+^ influx, via opening of receptor-operated calcium channels (ROCs). The proposed mechanism of inhibition mediated by Pp.Cr in term of its relaxant effect is summarized in Fig. 6.

## Conclusions

The aqueous ethanolic extract of Pyrus pashia (Pp.Cr) exhibited spasmolytic, bronchodilator and vaso-constrictive activities through different mechanisms. The spasmolytic and bronchodilator activities are likely to be mediated through blockade of Ca^2+^ channels, while vasoconstrictive activity may be due to presence of α-adrenergic, muscarinic, serotonergic and angiotensin II agonistic component. The Ca^2+^ channel blocking activitiy can be attributed to the phytochemical constituents of *Pyrus pashia* fruits (i.e., alkaloids, flavonoids, glycosides and anthraquinones).
